# Lectures and Observations on Clinical Surgery

**Published:** 1847-04-01

**Authors:** 


LECTURES AND OBSERVATIONS ON CLINICAL SURGERY. By *Andrew Ellis.* Small 8vo. pp. 275. Dublin, 1847.

ALTHOUGH the perusal of Mr. Ellis's little work impresses us with the belief that his Lectures must prove highly useful to those to whom they are addressed, yet they seem to us to scarcely contain sufficient of novelty or original observation to call for publication. The author manifests a disposition, however, to independently examine the foundations of received opinions, in spite of the prescriptive authority of great names by which these may be supported, which, tempered as it yet is by rendering of due deference to these, is well deserving of imitation and approval. We proceed to select a few passages from some of the Lectures.

*Treatment of Wounds of the Brachial Artery.*—After describing the various results of injury to this vessel, Mr. Ellis proceeds to give an account of their management, some portion of which is as follows:—

"Let us begin the consideration of this subject by asking what a surgeon ought to do, supposing he had reason to believe, from the history of the case and ocular evidence, that the brachial artery had been wounded? Under such circumstances, if the case were mine, I would proceed as follows:—In the first instance I would procure a long roller, and as much lint or sponge as would be sufficient to make a compress; the hemorrhage being restrained, in the mean time, by an assistant; who either with, or without the aid of a tourniquet made efficient pressure on the brachial artery above the wound; I would proceed to apply the bandage from the fingers (the fingers themselves being included) up to the bend of the arm: I would then place the compress over the wound, and with repeated figure of 8 turns of the bandage, make moderate pressure, with the hope that such a wound might heal without the supervention of any aneurismal affection whatever. I would then have the patient placed in bed, with his arm moderately extended on a pillow, and give directions that the bandage and compress should be kept constantly wet with a cold evaporating lotion. I would likewise adopt, in a rigid manner, the antiphlogistic plan of treatment, and moderate the heart's action by the cautious administration of small doses of tartar-emetic and digitalis. I have stated that I would keep the limb in the extended position; the reason why I consider this the best position is the following:—We are to suppose the wound in the artery is in the longitudinal direction, that is to say, that its greatest diameter corresponds with the long axis of the wounded vessel; this being the case, it is obvious that, by keeping the arm in the extended position, the extremities of the wound must be drawn far asunder; and in this way its lips or sides brought close to each other, and placed in a position favourable to union by adhesion. If the patient could bear this state of things for five or six days, I would then cautiously remove the bandage; but would not disturb the compress. I would now apply a new roller with a moderate degree of tightness, and direct a continuation of the antiphlogistic plan of treatment, with some mitigation, for at least a week longer. By this time the wound in the artery will, in all probability, have healed, and the liability to aneurism passed away. I have known two cases treated in this way with complete success; one by the late Mr. T. Roney, and the other by the late Mr. Todd, both gentlemen of high professional attainments and of well-merited distinction." P. 63.

This proceeding must, however, be discontinued if great pain and tension of the limb take place. Pressure having been thus tried in vain, and a diffused softish swelling attended with a "sort of undulatory pulsation" existing, we must open the tumour, remove the extravasated blood, and tie the artery above and below its wounded portion. In the case of *circumscribed* brachial aneurism the cure may be accomplished by compression or by the application of one ligature above the aneurismal tumour. The bandage already described should be applied with *moderate* tightness, great pressure not only being painful and sometimes dangerous by bursting the imperfectly formed sac, and converting the circumscribed into a diffused aneurism, but also in no-wise conducive to a cure. In the first Volume of the *Dublin Journal* is an account of three cases treated by Dr. Cusack by means of compression applied over the tumour, Valsalva's treatment being coincidently employed. In two, the cure was accomplished without causing the obliteration of the arteries engaged. In the third, the sac gave way, and the case was then treated by ligature. According to Scarpa's investigations the cure of these aneurisms is generally effected without obliteration of the artery or healing of the original wound in its parietes—the cured aneurisms he examined appearing to have degenerated into small tumours, consisting of the fibrine of the blood which had externally established a connexion with the circumjacent cellular membrane, and internally with the aperture of the artery. "In one of the cases the inner part of the aneurismal opening was closed by a calcareous deposit." Compression tried in vain, we must cut down and apply a ligature to the vessel without disturbing the aneurism.

"Gentlemen, we have now arrived at the consideration of the best method of treating the arterio-venous or varicose aneurism. It is a well established rule in surgery that we should never *operate* in a case of this kind unless when compelled to do so by urgent circumstances. For example, suppose pressure had been fairly tried without advantage, that the tumour is increasing in size, that it is painful and the limb swollen; when this state of things is established, the necessity for surgical interference is but too obvious. The operation which promises most advantage under such circumstances is that of tying the artery above the aneurismal tumour, as in the case I have just mentioned; leaving the tumour itself to the action of the absorbents. Judging from the literature of this subject, the operation should be undertaken with the utmost caution and circumspection, inasmuch as the records of surgery furnish but few cases in which it was not followed by untoward circumstances. I have myself witnessed but one case of this form of aneurism, in which the artery was tied. The case I allude to was treated by the late Mr. Hewson in the Meath Hospital. For some days after the operation the case appeared unpromising, the tumour having retained its former size and pulsation. On the third day after the operation Mr. Hewson applied a compress of lint over the tumour, and retained it in this position by strips of adhesive plaster, which were drawn obliquely over it; but in such a manner as not to completely encircle the limb, so as to impede the return of venous blood. In the course of three or four days, the compress was cautiously removed for the purpose of ascertaining the state of the tumour, which was most satisfactory. The swelling was not one-half the size it had been previous to the application of the compress: it had acquired a solid feel particularly at its circumference, and the pulsation had become exceedingly obscure. The compress was again applied as before: the ligature came away in eight or nine days, and the patient left the hospital in five or six weeks perfectly cured.

"In cases of aneurismal varix the surgeon should never interfere beyond recommending the patient to wear a bandage constantly on the limb to prevent over-distension of the veins." P. 73.

*Rules for the Employment of the Trephine.*—Mr. Ellis, after exhibiting the evils which resulted from the practice inculcated by Mr. Potts and his followers, states the limited number of circumstances under which the improvements of modern practice render this operation justifiable. 1. As it is a dangerous operation, it must not be undertaken unless it cannot render the patient's condition worse, or indeed unless it is likely to improve it. 2. It should be at once resorted to in the case of compound fracture, with depression, and bad constitutional symptoms. 3. It should be performed in a case of compound fracture, with depressed bone, and a foreign body, even although the constitutional symptoms be not urgent; for, in such a case, fatal inflammation may be afterwards set up, or the patient may become the subject of epilepsy or other diseased state of the brain. 4. Compound fracture with great depression, although there is no foreign body present, and the constitutional symptoms are slight, presents another case, but as eminent surgeons are here at issue, Mr. Ellis would not recommend interference in the case of children, unless the bone be depressed a full inch. We remember in the days of our pupillage, at St. Bartholomew's, nothing excited the deprecatory remarks of that formidable critical body, the Dressers, more than the apparent timidity which more than one of the surgeons manifested in meddling with this case of the category. Events, however, generally proved the correctness of their practice, and that in cases which were long watched after they had quitted the hospital. 5. In the case of contused wound of the scalp, especially if situated over the parietal bone, attended with fracture, without visible depression, but with bad constitutional symptoms, if reaction cannot be brought about by the usual means, rather than let the patient die without the chance, the trephine should be used in the possibility of the pressure being caused by the depression of the inner table, or the effusion of blood upon the dura mater. 6. In contused wound of the scalp without visible fracture, but with very urgent constitutional symptoms, the same reasoning is employed by Mr. Ellis. 7. The same in violent contusion of the scalp, with bad constitutional symptoms. 8. In the case of recurring insensibility after temporary recovery from the effects of a blow on the head, the operation is always indicated, the compression arising from effused blood. 9. It is so likewise when the history and symptoms lead to the belief that matter has formed between the dura mater and skull, although it is seldom of service, in consequence of the deeper-seated portions of the organ being simultaneously affected. 10. The presence of a sinus must never prevent the operation, providing the cause of compression is there located.

It will be seen that Mr. Ellis admits of a greater extension of this operation than is generally now done. Much may be said, drawn from the desperate nature of these cases, in favour of such practice; but we believe that the instances of success are not sufficiently numerous to offer much encouragement.

*Rupture of the Bladder.*—"In the first place, it may, perhaps, appear somewhat strange to you, that the bladder should have given way, in both instances, in that part which is covered by peritoneum. Now you are not to consider this in the light of an accidental circumstance; in every case I am acquainted with, in which the bladder gave way, in consequence of falls or blows on the abdomen, the rupture took place in the peritoneal region of the organ. In support of this statement, I beg to refer you, to two very important cases of this description, which have been published in the second volume of the '*Dublin Hospital Reports*,' by Dr. Cusack; and also, to the ninth volume of the '*Dublin Journal of Medical Science*,' in which you will find the particulars of some interesting cases, detailed by my distinguished colleague, Dr. Harrison. The only explanation I would venture to suggest, is the anatomical fact, that the superior and posterior regions are weaker than the other parts of the bladder; inasmuch as, they do not receive any support from the reflections of the pelvic fasciæ

whilst the peritoneal covering, which is comparatively thin and delicate, and being, in common with all serous membranes, devoid of elasticity, is therefore, incapable of accommodating itself to violence suddenly applied, and yields only by the laceration of its proper structure.

"The next point to which I am anxious to call your attention, is the difference which exists between the local consequences which result from urinary extravasations into the cellular, and the serous tissues. I have already stated, that inflammation and mortification are the usual effects, when they take place in the cellular membrane; now, I wish you to understand, that, in no instance with which I am acquainted, did mortification ensue from urinary extravasation into the cavity of the peritoneum. Judging from the usual effects of injury and irritation, when applied to this very delicate and sensitive membrane, it appears strange, that in cases of ruptured bladder, in which the peritoneum is not only wounded, but brought in contact with a very acrimonious fluid, that it should be slow in taking on inflammatory action, and capable of resisting its gangrenous consequences." P. 206.

Mr. Ellis appends to his Clinical Lectures a discourse which he delivered elsewhere on Hydrophobia, which contains a highly interesting summary of the leading facts bearing upon the subject. Appended to it is a collection of twelve cases, having for object to prove "*that a tame or healthy dog may produce hydrophobia in the human subject.*" In eight of these, collected from various sources, the dog gave no prior or subsequent evidence of rabidity, and yet communicated fatal disease. "These cases ought, in my mind, be deemed sufficient to convince the greatest sceptic, that the bite of a dog, which *is not rabid,* can produce hydrophobia in the human species!" Four other cases are cited in affirmation of another query, viz. "can a dog in perfect health, in a state of tranquillity, and when it does not bite or attempt to bite, produce hydrophobia in the human subject?" In these the animal communicated the disease by licking the face or some abraded surface. The cases alluded to are not sufficiently particularized and authenticated to carry entire conviction to our minds: but we fully believe Mr. Ellis has here brought a most important question under the notice of the public, and one to which sufficient attention has not been hitherto paid.

